# Novel nanomedicine with a chemical-exchange saturation transfer effect for breast cancer treatment in vivo

**DOI:** 10.1186/s12951-019-0557-0

**Published:** 2019-12-17

**Authors:** Yanlong Jia, Chaochao Wang, Jiehua Zheng, Guisen Lin, Dalong Ni, Zhiwei Shen, Baoxuan Huang, Yan Li, Jitian Guan, Weida Hong, Yuanfeng Chen, Renhua Wu

**Affiliations:** 1 Department of Radiology, Second Affiliated Hospital, Shantou University Medical College, Shantou, 515041 People’s Republic of China; 2 Shanghai Key Laboratory of Advanced Polymeric Materials, School of Chemistry and Molecular Engineering, East China University of Science and Technology, 130 Meilong Road, Shanghai, 200237 People’s Republic of China; 3 Department of General Surgery, Second Affiliated Hospital, Shantou University Medical College, Shantou, 515041 People’s Republic of China; 4 Departments of Radiology and Medical Physics, University of Wisconsin-Madison, Madison, WI 53705 USA

**Keywords:** Breast cancer, Magnetic resonance imaging, Chemical exchange saturation transfer, Doxorubicin, Nanomedicine

## Abstract

**Background:**

Nanomedicine is a promising new approach to cancer treatment that avoids the disadvantages of traditional chemotherapy and improves therapeutic indices. However, the lack of a real-time visualization imaging technology to monitor drug distribution greatly limits its clinical application. Image-tracked drug delivery is of great clinical interest; it is useful for identifying those patients for whom the therapy is more likely to be beneficial. This paper discusses a novel nanomedicine that displays features of nanoparticles and facilitates functional magnetic resonance imaging but is challenging to prepare.

**Results:**

To achieve this goal, we synthesized an acylamino-containing amphiphilic block copolymer (polyethylene glycol-polyacrylamide-polyacetonitrile, PEG-b-P(AM-*co*-AN)) by reversible addition-fragmentation chain transfer (RAFT) polymerization. The PEG-b-P(AM-*co*-AN) has chemical exchange saturation transfer (CEST) effects, which enable the use of CEST imaging for monitoring nanocarrier accumulation and providing molecular information of pathological tissues. Based on PEG-b-P(AM-*co*-AN), a new nanomedicine PEG-PAM-PAN@DOX was constructed by nano-precipitation. The self-assembling nature of PEG-PAM-PAN@DOX made the synthesis effective, straightforward, and biocompatible. In vitro studies demonstrate decreased cytotoxicity of PEG-PAM-PAN@DOX compared to free doxorubicin (half-maximal inhibitory concentration (IC50), mean ~ 0.62 μg/mL vs. ~ 5 μg/mL), and the nanomedicine more efficiently entered the cytoplasm and nucleus of cancer cells to kill them. Further, in vivo animal experiments showed that the nanomedicine developed was not only effective against breast cancer, but also displayed an excellent sensitive CEST effect for monitoring drug accumulation (at about 0.5 ppm) in tumor areas. The CEST signal of post-injection 2 h was significantly higher than that of pre-injection (2.17 ± 0.88% vs. 0. 09 ± 0.75%, *p* < 0.01).

**Conclusions:**

The nanomedicine with CEST imaging reflects the characterization of tumors and therapeutic functions has great potential medical applications.

## Background

Many small-molecule drugs are widely used to treat malignant tumors. Doxorubicin (DOX), an important anthracycline antibiotic, is a broad-spectrum and aperiodic specific anticancer drug with wide clinical applications against various malignancies, including breast cancer, soft tissue sarcomas, and hematological malignancies [1–3]. DOX exerts its anticancer effects by breaking the intracellular DNA chain to prevent the DNA replication, transcription, and macromolecular biosynthesis processes, ultimately leading to cancer cell death [4]. Although DOX shows high cytotoxicity against cancer cells, its clinical utility is limited owing to its rapid clearance from the body, poor target selectivity, chemoresistance, and serious side effects [5]. Hence, to restore the clinical effectiveness of DOX against cancer, innovative technologies and methods are needed.

Recent developments of nano-theory and technology have resulted in various novel drug delivery systems [6, 7] such as liposomes [8–10], polymeric nanomicelles [11], metal nanoparticles (NPs) [12], inorganic NPs [13], and mesoporous silica [14]. Drug-loaded nanocarriers are small in size and are therefore easily absorbed by cells [15]; moreover, they preferentially accumulate in tumors owing to the enhanced permeability and retention (EPR) effect [16]. The drugs are then released and kill the cancer cells. An ideal drug nanocarrier should have high water solubility, high endocytosis efficiency, low cost, low cytotoxicity toward normal cells, and a long circulation time. Amphiphilic block copolymer NPs are common types of nanomicelles that have recently come into the research spotlight given their following advantages: their surfaces can be easily modified, they show good biocompatibility, have a long plasma half-life, are of low toxicity, are associated with lower costs, and are environmentally friendly [17–19]. Polymeric NPs are mainly composed of amphiphilic block copolymers with hydrophobic and hydrophilic fragments, which form a hydrophobic core-hydrophilic shell structure by self-assembly in a selective solvent [20]. Thus, polymer NPs can load hydrophobic small-molecule drugs on their core, thereby improving the solubility of drugs. Drug stability is enhanced through interactions between the hydrophobic ends. In addition, the plasma half-life is prolonged because the hydrophilic ends are not easily recognized by the defense system [21]. Therefore, an NPs formulation could be a promising means for reducing the systemic toxicity of traditional chemotherapy and improving therapeutic indices.

Along with good stability, it is important that analysis of the distribution of a drug in the circulation is possible. Image-guided delivery of nanomedicines in vivo is of great clinical interest, as it can help identify patients for whom the treatment is more likely to be beneficial, which is particularly important for establishing tailored individualized treatments. To date, various imaging techniques have been studied for their ability to track the delivery of drugs in vivo. However, each in vivo imaging modality has its own limitations. For example, owing to its low sensitivity and poor capability of revealing biochemical or physiological abnormalities, the extensive application of magnetic resonance imaging (MRI) may be limited [22]. Gadolinium-enhanced T1WI and dynamic contrast enhancement MRI require injection of contrast agents, which increase the risks of possible Gd accumulation in the tissue and renal fibrosis [23]. F18-fluorine-2-deoxy-d-glucose positron emission tomography can provide information regarding energy metabolism in the early stage of tumor formation [24]; however, this technique also has low specificity, high costs, and requires injection of radioactive substances [25]. Magnetic resonance spectroscopy is limited by its relatively poor detection sensitivity and poor spatial resolution in vivo [26, 27]. Moreover, the wide application of optical imaging is hindered by its intrinsic depth limitation [28]. Thus, novel methods are needed to allow for nanomedicines to be tracked in vivo after their administration. Visualizing their accumulation in tumors would facilitate evaluations of disease progression more comprehensively and enable more accurate predictions of tumor progression.

Chemical exchange saturation transfer (CEST) MRI is a novel contrast mechanism that allows for the amplified detection of low-concentration molecules by applying selective radiofrequency (RF) saturation pulses on exchangeable protons [29–31]. The saturated exchangeable protons then exchange with bulk water protons, resulting in partial loss of the bulk water signal, which then becomes detectable during MRI [32]. CEST MRI can be switched “on” and “off” at will by simply adjusting the RF saturation pulse sequence parameters [33]. Notably, CEST MRI has potential to provide molecular information for diagnosing pathological tissues and detecting molecular responses to treatment [34, 35]. Moreover, nanoscale carriers could be used as a CEST contrast agent to detect substances at very low concentrations (i.e., at the micromolar or nanomolar scale) [36]. Most importantly, NP-based CEST contrast agents can be specifically tailored to respond to a given stimulus (e.g., pH, enzyme), with benefits for imaging sensitivity and specificity [37, 38]. It is thus possible to extend CEST technology to the nano-technology realm through integrating CEST contrast agents into nanocarriers.

Therefore, we aimed to synthesize a novel nanomedicine using DOX that could not only overcome the drawbacks of traditional chemotherapy but would also allow for detection in the circulation by CEST.

## Materials and methods

### Acrylamide (AM)

Acrylamide (AR, 99.0%; Aladdin Biochemical Technology Co., Ltd., Shanghai, China) is a small-molecule compound with a molecular weight of 71.08, and was prepared at different concentrations (10 mM, 30 mM, 50 mM, and 100 mM) at the same pH of 7.8 for CEST scanning. To evaluate whether the CEST effect of AM is pH-dependent, solutions of four different pH (7.2, 7.4, 7.6, and 7.8) were titrated at the same concentration (50 mM). Different saturation powers (0.5–4.0 μT) and saturation times (1–5 s) were also used to find the optimized conditions. All imaging procedures conducted in this study were performed on an Agilent 7.0 T MR system (Agilent Technologies, Santa Clara, CA, USA) with a standard 9563 body coil for signal transmission and reception. For in vitro experiments, an improved version of continuous wave echo planar imaging sequence (CW-EPI) [39] was used with the following parameters: TR = 6000 ms, TE = 29.46 ms, Kzero = 32, slice thickness = 2 mm, FOV = 30 × 30 mm, matrix size = 64 × 64. The total imaging duration was 613 s.

### Synthesis of PEG-b-(PAM-*co*-PAN) and PEG-PAM-PAN@DOX

The amphiphilic block copolymer polyethylene glycol (PEG)-*b*-(PAM-*co*-PAN) was synthesized using a PEG-based macro-RAFT. AM (40 mmol, 2.83 g), acrylonitrile (10 mmol, 0.52 g), azodiisobutyronitrile (AIBN, 0.002 mmol, 0.33 mg), PEG-RAFT (0.02 mmol, 0.11 g), and 5 mL dimethyl sulfoxide (DMSO) were added to a 25-mL reaction flask equipped with a magnetic stir bar and a rubber seal. The air of the reaction flask was removed by vacuuming, and argon was injected and circulated three times. The polymerization was performed at 65 °C for 12 h in an oil bath. The polymerization was terminated by exposure to air. The product was precipitated into diethyl ether, and this process was repeated three times. The final product was dried in vacuum at 30 °C for 48 h, yielding a white solid (2.81 g, yield: 81.2%, *M*_*n,GPC*_ = 37,982*, Mw/Mn* = 1.32). ^1^H-NMR (400 MHz, *d*_6_-DMSO, δ):1.24 (m, –CH_3_), 1.80–2, 32(–CH–CH_2_–), 3.51 (–OCH_2_CH_2_O–), 6.95–7.46 (–CONH_2_).

**Scheme 1:**
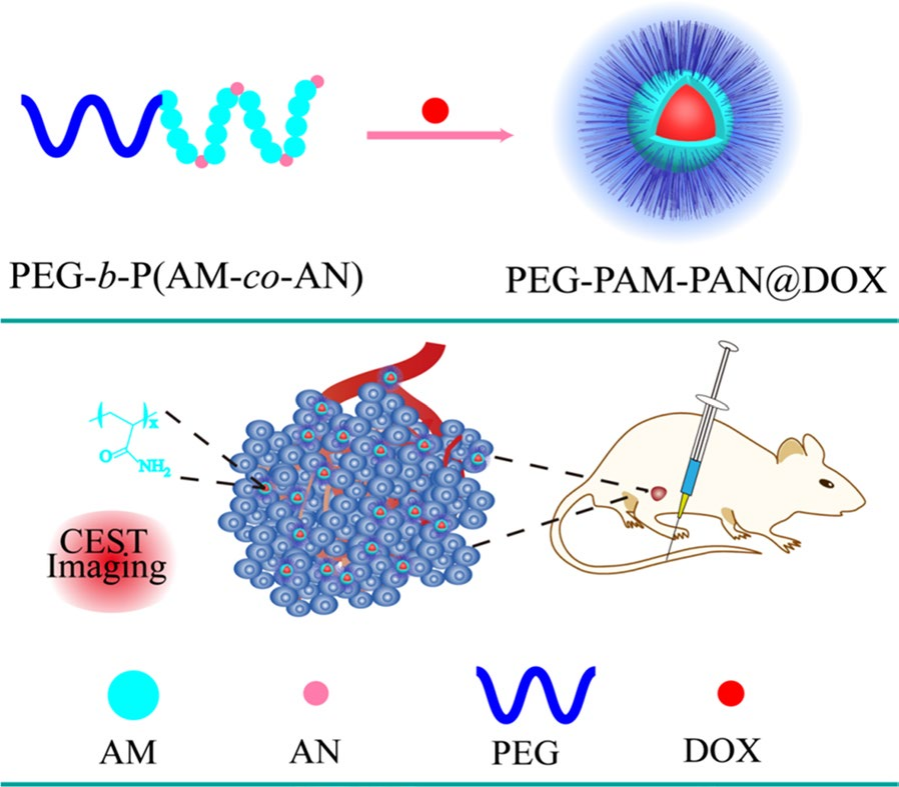
Schematic diagram of the fabrication of PEG-PAM-PAN@DOX for chemotherapy and CEST imaging

The typical fabrication process of PEG-PAM-PAN@DOX is shown in Scheme 1. In brief, 4 mg of DOX powder and 20 mg of the PEG-PAM-PAN block copolymer were dissolved together in 2 mL of DMSO, which was added to 8 mL of deionized water upon stirring. DMSO was then removed by dialysis (MWCO = 12,000 Da) against deionized water for 24 h, and fresh deionized water was replaced every 2 h to ensure complete removal of excess DOX molecules that failed to be entrapped by the polymer NPs. The polymer NPs were concentrated by ultrafiltration. The final concentration of PEG-PAM-PAN@DOX was 10 mg/mL.

### Characterization

The particle size and morphology of the NPs were characterized by dynamic light scattering (DLS) on a Nano-Zetasizer system (Malvern Instruments Ltd.) and transmission electron microscopy (TEM) analysis performed on a JEOL 200CX microscope with an accelerating voltage of 200 kV. MDA-MB-231-Luciferase-Pur breast cancer cells (Fubio Biochemical Technology Co., Ltd., Shanghai, China) were used in this study for evaluation, which can be widely used for in vivo bioluminescent assays. MDA-MB-231-Luciferase-Pur breast cancer cells (10 × 10^4^ cells/well) were cultured overnight in a laser confocal glass plate. After adherent growth, the medium containing the NPs replaced the original culture medium and the cells were grown for 2 h or 24 h. The culture medium was discarded and washed with phosphate-buffered saline (PBS) thrice to remove the free NPs that were not uptaken by MDA-MB-231-Luciferase-Pur cells. Finally, the sample was fixed using a paraformaldehyde solution (40 g/L) for 30 min and washed with PBS thrice. The cells were stained using 4-6-diamidino-2-phenylindole (DAPI) for 5 min and again washed with PBS thrice. Confocal microscopy was used to observe the sample under an oil lens. DAPI stained the nuclei blue, DOX emitted red fluorescence, and the fusion of DAPI and DOX was observed as purple fluorescence.

### Toxicity assessment

#### Cytotoxicity assessment

The breast cancer cells were cultured at 37 °C with 5% CO_2_ in Dulbecco’s modified Eagle’s medium (DMEM) supplemented with 10% fetal bovine serum and 1% streptomycin/penicillin. The cells were seeded into a 96-well cell culture plate at 10^4^ cells/well, which were then incubated for 24 h at 37 °C under 5% CO_2_. DMEM solutions of PEG-PAM-PAN with different concentrations (0.8, 1.6, 3.1, 6.2, 12.5, 25, 50, 100, and 200 μg/mL) were added to the wells. Different concentrations (0.31, 0.62, 1.25, 2.5, 5 and 10 μg/mL) of free DOX and PEG-PAM-PAN@DOX were also added to the wells to measure the cell viability, calculated using a typical methyl thiazolyl tetrazolium (MTT) assay.

#### In vivo toxicity assessment

Fifteen Kunming mice (Laboratory Animal Center, Shantou University Medical College) with an average weight of 20 g were used for in vivo toxicity evaluation. The mice were divided into three groups: group 1 (control) mice were injected with saline only, whereas mice in group 2 and group 3 were administered PEG-PAM-PAN once via tail intravenous injection at a total dose of 10 mg/kg and observed for 7 days and 30 days, respectively. The survival and body weight of the mice were evaluated every 3 days. Tissue and blood samples were collected from mice of all three groups. Hematoxylin and eosin (H&E) staining of the heart, liver, spleen, lungs, and kidney tissues of the mice was performed. Four important hepatic indicators (alanine aminotransferase [ALT], aspartate aminotransferase [AST], alkaline phosphatase [ALP], and total protein [TP]), three indicators of kidney functions (creatinine [CRE], blood urea nitrogen [BUN], and urea [UA]), and complete blood count (CBC) were determined.

### Chemotherapeutic efficacy model

All animal care and experimental procedures were approved by the Animal Care and Use Committee of Shantou University Medical College (Approval ID: SUMC2019-179) and were in accordance with the National Research Council's Guide for the Care and Use of Laboratory Animals. For this assessment, 20 5-week-old female BALB/c nude mice (Beijing Vital River Laboratory Animal Technology Co., Ltd.), weighing 18−22 g, were used, which were maintained at the Laboratory Animal Center of Shantou University Medical College. All mice were kept in a specific pathogen-free animal room with a temperature-controlled system and a 12-h dark–light cycle. They were fed standard laboratory diet and water. The animals were acclimatized to the environment for 1 week before the experiment.

Approximately 2 × 10^6^ MDA-MB-231-Luciferase-Pur breast cancer cells were implanted into the fourth left mammary fat pad (n = 12) and inoculated subcutaneously into the right hind limb (n = 8) of the 6-week-old female BALB/c nude mice. Tumors were allowed to grow for 2 to 3 weeks, until they were approximately 5 mm in diameter. The mice were divided into three groups of a control group, DOX group (5 mg/kg), and PEG-PAM-PAN@DOX group (3 mg/kg DOX). As the substrate of firefly Luciferin, VivoGlo Luciferin, can glow in a tumor model of breast cancer expressing luciferase, tumor growth was observed 10 min after intraperitoneal injection (150 mg/kg) with an in vivo fluorescence imager (IVIS Kinetic). Twelve orthotopically xenografted tumors were evaluated in the three groups with four animals per group. Tumor size and the body weight of mice were measured every 3 days from day 0 to the day of euthanasia (day 21) using a Vernier caliper and electronic scale, respectively. The volume of the tumor was calculated using the following formula: $\text{V}=\text{a} \times {\text{b}^2}/2$ (a is the longest diameter of the tumor, while b is the longest diameter perpendicular to a). The relative volume was calculated by comparing the final volume to the initial tumor volume. H&E and immunohistochemical staining was performed to reveal the changes in the tumors at the cellular level. Eight tumors in the subcutaneous tissue of the right hind limb were scanned for CEST imaging before, and 30 min, 1 h, 2 h, and 2.5 h after intravenous injection of 200 μL PEG-PAM-PAN@DOX (~ 10 mg/mL NPs) to detect drug accumulation.

### In vivo CEST imaging

The mice were anesthetized with isoflurane vaporized with 5% O_2_; 4.0% isoflurane was used for anesthesia induction and 2.0–2.5% isoflurane was used for maintenance. The breath rate was monitored throughout the MRI experiments using a respiratory probe. The tumors were positioned at the isocenter of the magnet for optimal shimming. To eliminate signal interference of B_0_ field inhomogeneity, the B_0_ map was shimmed prior to the experiments with the following parameters: TR = 40 ms, TE = array, slice thickness = 4 mm, FOV = 25 × 25 mm, matrix size = 64 × 64, flip angle = 15°, averages = 12. A high-resolution T2-weighted axial slice crossing the center of the tumors was acquired with TR = 4000 ms, TE = 10 ms, slice thickness = 2 mm, FOV = 30 × 30 mm, matrix size = 128 × 128, segments/ETL = 16/8, Kzero = 4. For in vivo CEST imaging, the parameters were as follows: TR = 6000 ms, TE = 27.63 ms, slice thickness = 4 mm, FOV = 25 × 25 mm, matrix size = 64 × 64, ETL = 64, Kzero = 32, shots = 1, repetitions = 1, averages = 1, dummy scans = 7, with 122 frequency offsets unevenly distributed from − 6 to 6 ppm relative to the resonance of water. The total scanning duration was 793 s.

### Image processing and data analysis

All CEST image processing and data analysis were performed using custom-written scripts in MATLAB (Mathworks, Natick, MA, USA, R2011b). The Water Saturation Shift Reference (WASSR) method was used to correct for B_0_ field inhomogeneity [40]. Regions of interest were drawn manually based on the T2-weighted images covering the entire tumor. Saturation transfer efficiency (ST %) was measured by magnetization transfer ratio (MTRasym), which was defined by the following expression:

$$\text{MTRasym}=(\text{S-}\Delta\upomega-\text{S+}\Delta\upomega)/{\text{S}_0} \,\text,$$

where S sat (+ Δω) and S sat (− Δω) are the signal intensities obtained by saturating at the frequency of Δω downfield and up field from the water proton resonance frequency. S_0_ is the water signal intensity in the absence of the saturation pulse. The MTRasym data were tested using paired *t*-tests between pre-injection and post-injection scans, and the other statistical significance data were analyzed using a standard analysis of variance (ANOVA). Statistical evaluations were performed using GraphPad Prism software with a significance level of *p* < 0.05.

## Results and discussion

### In vitro CEST imaging of AM

**Fig. 1:**
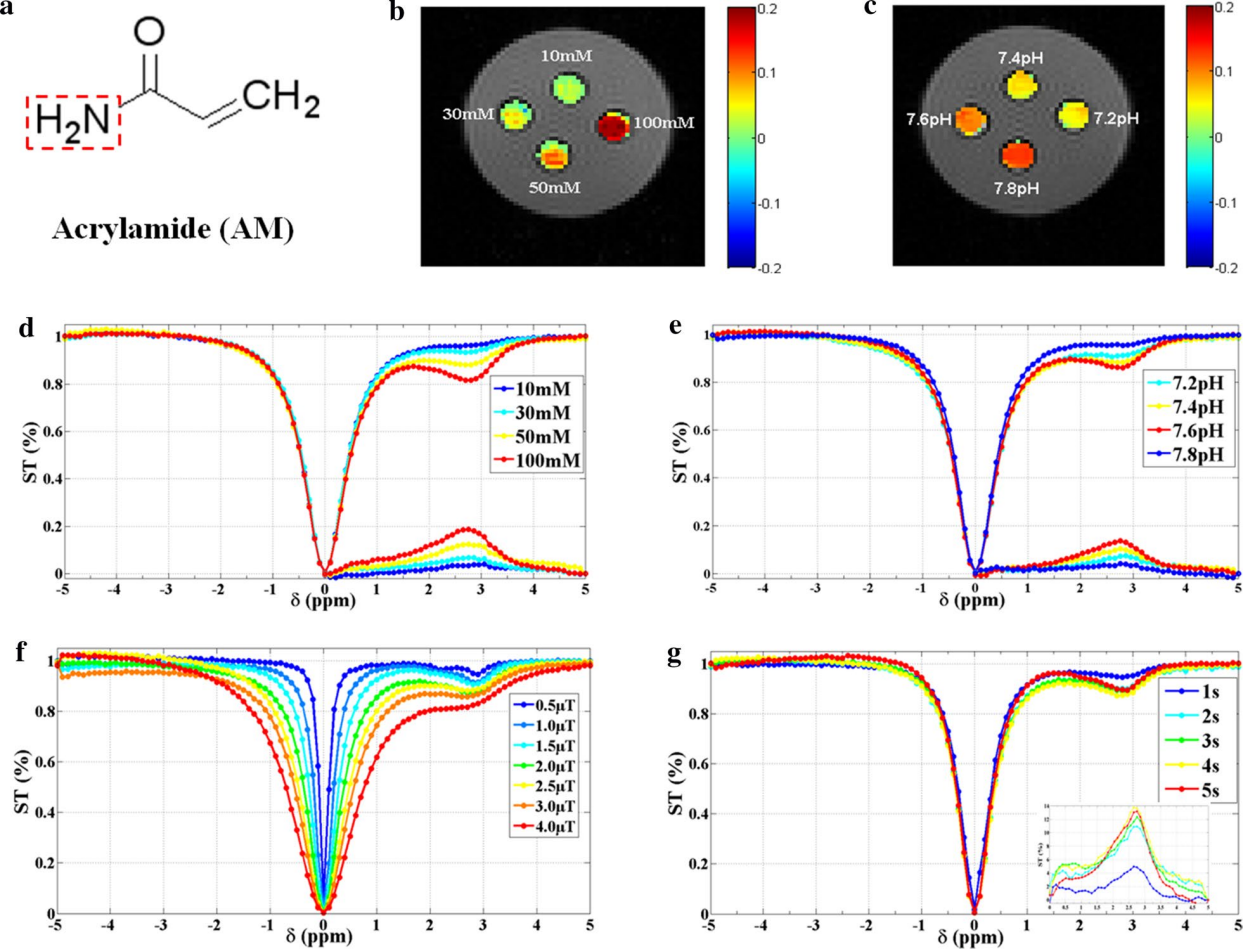
In vitro CEST imaging and Z-spectra of AM at different scanning parameters. **a** The chemical structure of AM; **b**, **c** CEST imaging of AM at different concentration and pH showed that the CEST effects of AM were concentration and pH-dependent; **d**, **e** Z-spectra of AM at different concentration and pH revealed that the clear CEST effect peaked at approximately 2.75 ppm; **f**, **g** Z-spectra of AM at different saturation power (μT) and saturation time (s) showed that the CEST effects were increased with the saturation power and time within a certain range. Colors bar represented the signal intensity

The amine protons on AM generated a CEST effect with selective saturation at 2.75 ppm (Fig. 1). To our knowledge, this represents the first demonstration of this CEST effect of AM. Figure 1b, d show that the CEST signal of AM was concentration-dependent with the ST% increasing from 3.03% (10 mM) to 19.58% (100 mM). Similarly, the CEST signal of AM was also pH-dependent with the ST% increasing from 5.93% at pH 7.2 to 13.36% at pH 7.8 (Fig. 1c, e); thus, the optimal pH was determined to be 7.8. This is consistent with a previous study showing that amide proton exchange is base-catalyzed [41]. The observed CEST spectrum depends on the imaging parameters as well as on the underlying tissue microenvironment [35, 39, 40]. Therefore, we optimized the CEST parameters, including pulse duration and saturation power of the irradiation RF pulse on a phantom. These results demonstrated that the CEST effect increased as the saturation power and saturation time increased within a certain range (Fig. 1f, g). The optimal saturation power and time of AM peaked at 3.0 μT and 4 s, respectively (Additional file 1: Figure S1). Balaban and co-workers have only been able to detect small molecules at 50–100 mM concentrations [42, 43]. However, after optimization, we could directly detect small molecules (AM) at much lower concentrations (10 mM) under a high magnetic field of 7.0 T. Nevertheless, this concentration is still too high for clinical applications. NPs have been shown to enhance CEST sensitivity since they possess a large amount of exchangeable protons [44]. Hence, adopting a nanotechnology approach would allow for incorporating a large number of AM into a well-defined nanostructure to improve the CEST sensitivity.

### Synthesis and characterization of PEG-PAM-PAN@DOX

**Fig. 2:**
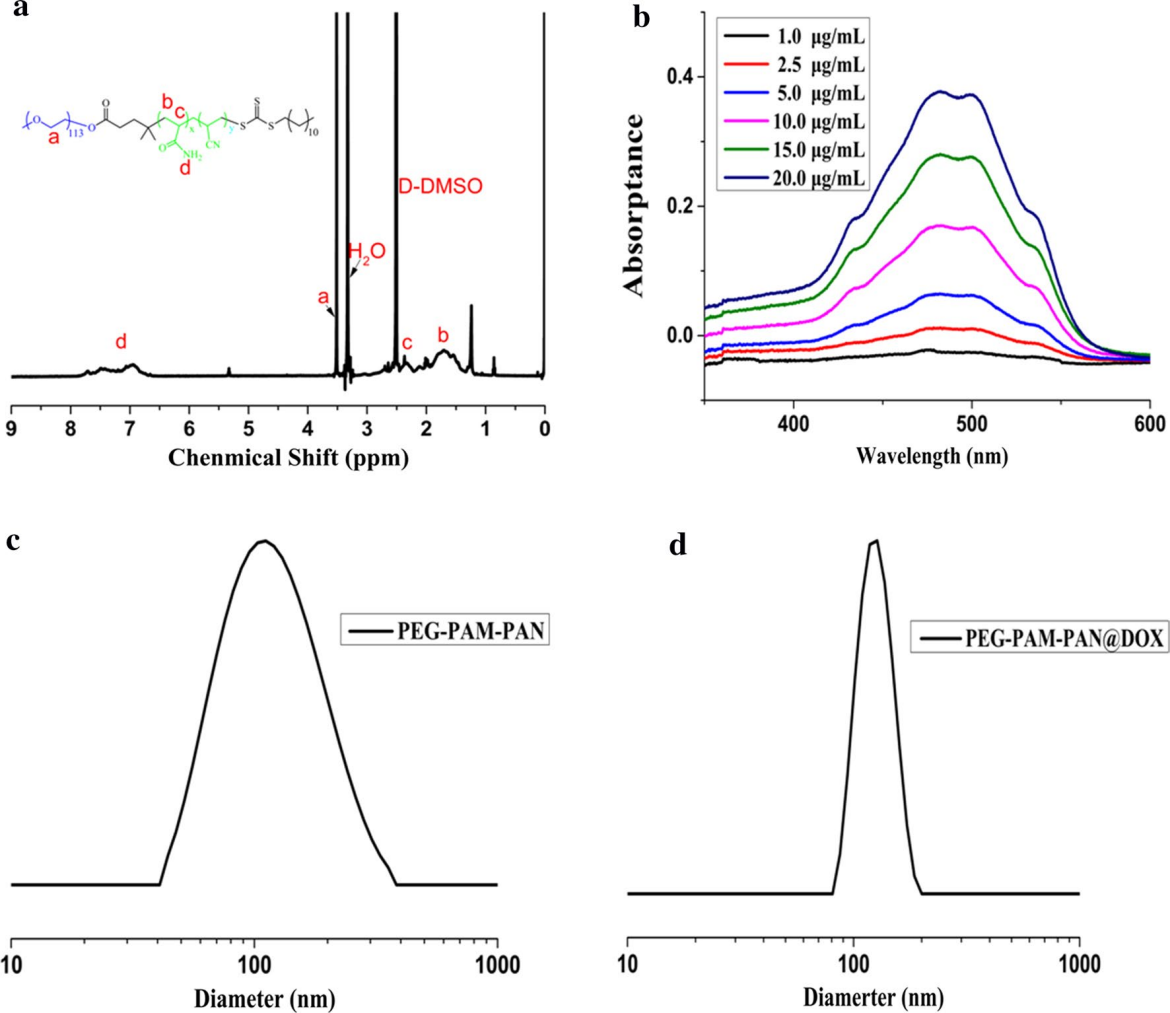
The basic manifestations and standard curve of nanoparticles. **a** Nuclear magnetic spectra of compound PEG-PAM-PAN indicated that the self-assembly was successful; **b** UV–Vis spectra of DOX solution in PBS with different concentration and the absorptance of DOX was concentration-dependent; **c**, **d** The particle size of PEG-PAM-PAN and PEG-PAM-PAN@DOX was 113.4 nm (PDI = 0.241) and 127.2 nm (PDI = 0.152) characterized by DLS

The detailed structure of PEG-PAM-PAN@DOX is shown in Additional file 1: Figure S2. Uniform PEG-b-P(AM-*co*-AN) NPs were synthesized using a self-assemblage method, and nuclear magnetic spectra of PEG-PAM-PAN indicated that the self-assembly was successful (Fig. 2a). Based on PEG-b-P(AM-*co*-AN), a new nanomedicine, PEG-PAM-PAN@DOX, was fabricated by the nano-precipitation method. TEM images showed that both the PEG-PAM-PAN and PEG-PAM-PAN@DOX NPs were well-dispersed, spherically shaped particles, which were stable in water and did not form aggregates owing to their core–shell structure (Additional file 1: Figure S3). Ultraviolet spectrophotometry, taking the DOX concentration as the horizontal coordinate and absorbance value as the vertical coordinate (Fig. 2b), demonstrated a good linear relationship with the regression equation $\text{Y}=0.02117*\text{X}-0.0423$ (R^2^ = 0. 9998) (Additional file 1: Figure S4). This linear regression equation was then used to calculate the amount of DOX loaded in the NPs. DLS was performed to characterize the particle size distribution of PEG-PAM-PAN and PEG-PAM-PAN@DOX NPs, demonstrating an average particle diameter of 113.4 nm (PDI = 0.241) and 127.2 nm (PDI = 0.152), respectively (Fig. 2c, d).

### In vitro CEST imaging of PEG-PAM-PAN@DOX

As mentioned above, the magnitude of the CEST signal depends on the number of exchangeable protons [22, 32]. Detection of a small-molecule compound is therefore generally only possible at high concentrations; however, drugs are not typically present at such high concentrations in vivo. Therefore, it has been necessary to label these compounds with NPs endowed with many exchangeable protons.

**Fig. 3:**
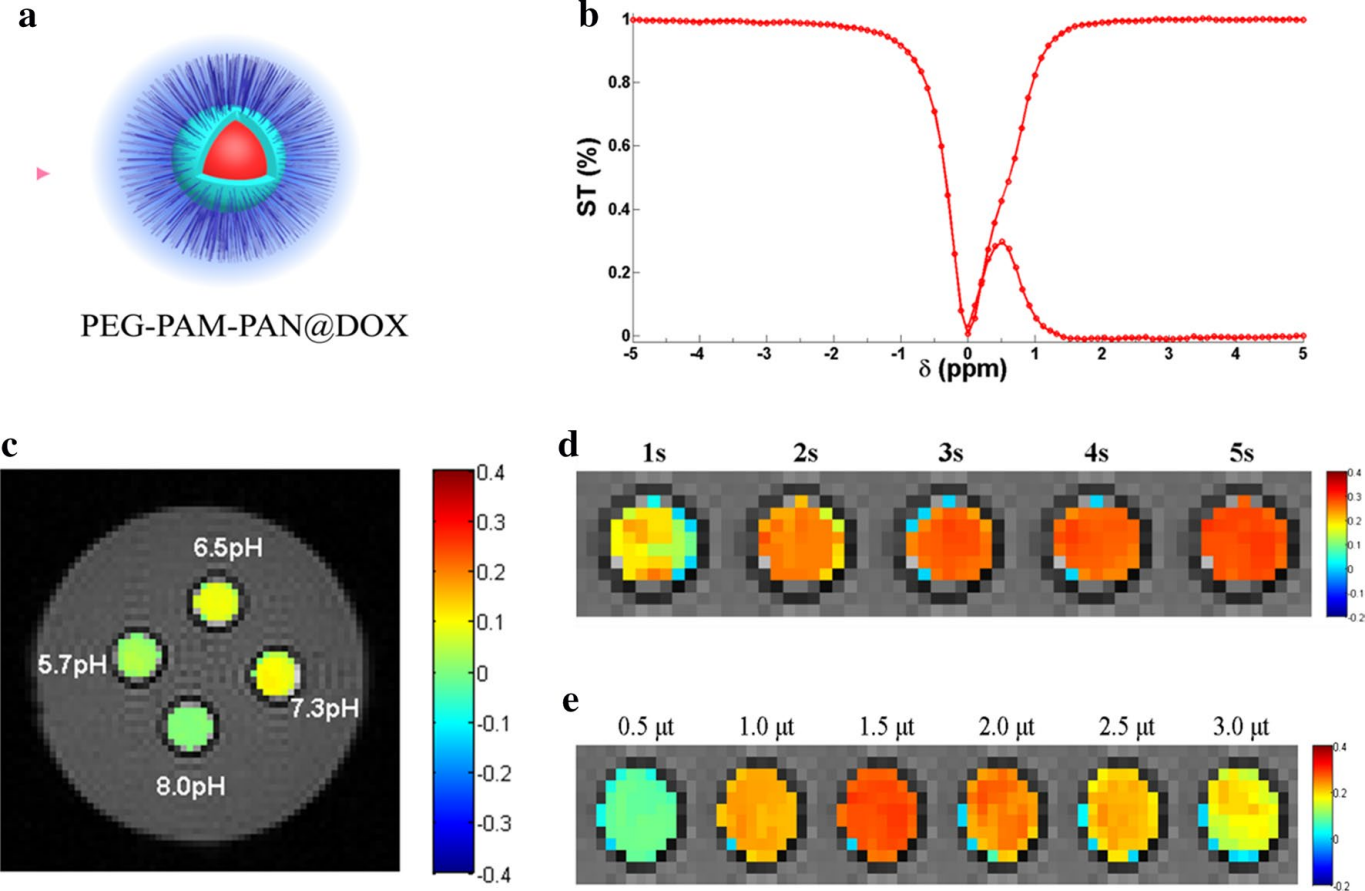
In vitro CEST imaging and Z-spectra of PEG-PAM-PAN@DOX. **a** Cartoon depicting PEG-PAM-PAN@DOX NPs; **b** Z-spectra of PEG-PAM-PAN@DOX showed that the CEST effects were at approximately 0.5 ppm; **c** CEST imaging of PEG-PAM-PAN@DOX at different pH; **d** CEST imaging of PEG-PAM-PAN@DOX at different saturation time (s) and that these increased with the saturation time; **e** CEST imaging of PEG-PAM-PAN@DOX at different saturation power (μT) and the peaked at 1.5 μT

The Z-spectra in Fig. 3b show a noticeable saturation transfer effect for PEG-PAM-PAN@DOX at 0.5 ppm, which represents a distinct shift different from that of the monomer AM. P(AM-*co*-AN) is a temperature-responsive polymer with an upper critical solution temperature (UCST) [45]. When the temperature is lower than the UCST, the interaction between P(AM-*co*-AN) and H_2_O is reduced and P(AM-*co*-AN) is almost insoluble in water. CEST imaging is based on H^+^ exchange between the detection compound and water; therefore, the interaction between P(AM-*co*-AN) and water may affect the chemical shift of AM on PEG-PAM-PAN@DOX in CEST imaging. The temperature in our experiment was lower that the UCST of PEG-b-P(AM-*co*-AN), which could explain why PEG-PAM-PAN@DOX was detected at 0.5 ppm during CEST imaging.

For the in vitro experiment, we adjusted the pH of the solutions to 5.7, 6.5, 7.3, and 8.0 to observe the CEST effect of PEG-PAM-PAN@DOX (Fig. 3c). The CEST signal decreased at a pH of 5.7 (~ 3.42%) and 8.0 (~ 0.57%). Therefore, a weak acid (6.5 pH, ~ 8.67%) or neutral pH (7.3 pH, ~ 9.15%) was more suitable to observe the CEST signal of PEG-PAM-PAN@DOX. In addition, the CEST effect of PEG-PAM-PAN@DOX increased as the saturation power or time increased, which was consistent with findings of a previous study [46]. Nevertheless, the CEST effect could not be improved further when the saturation power was above a certain threshold (Fig. 3e). The duration of the saturation pulse was also critical for an optimal CEST effect (Fig. 3d). The CEST effect was positively correlated with the saturation time, and the peak was observed at 5 s (Additional file 1: Figure S5). A long saturation time can be advantageous for in vivo CEST by minimizing the saturation power [47, 48]. Thus, based on experiments on a phantom, the optimal saturation power (1.5 μT) and long saturation time (5 s) were selected for the subsequent in vivo CEST imaging experiments.

### Toxicity and uptake studies

**Fig. 4:**
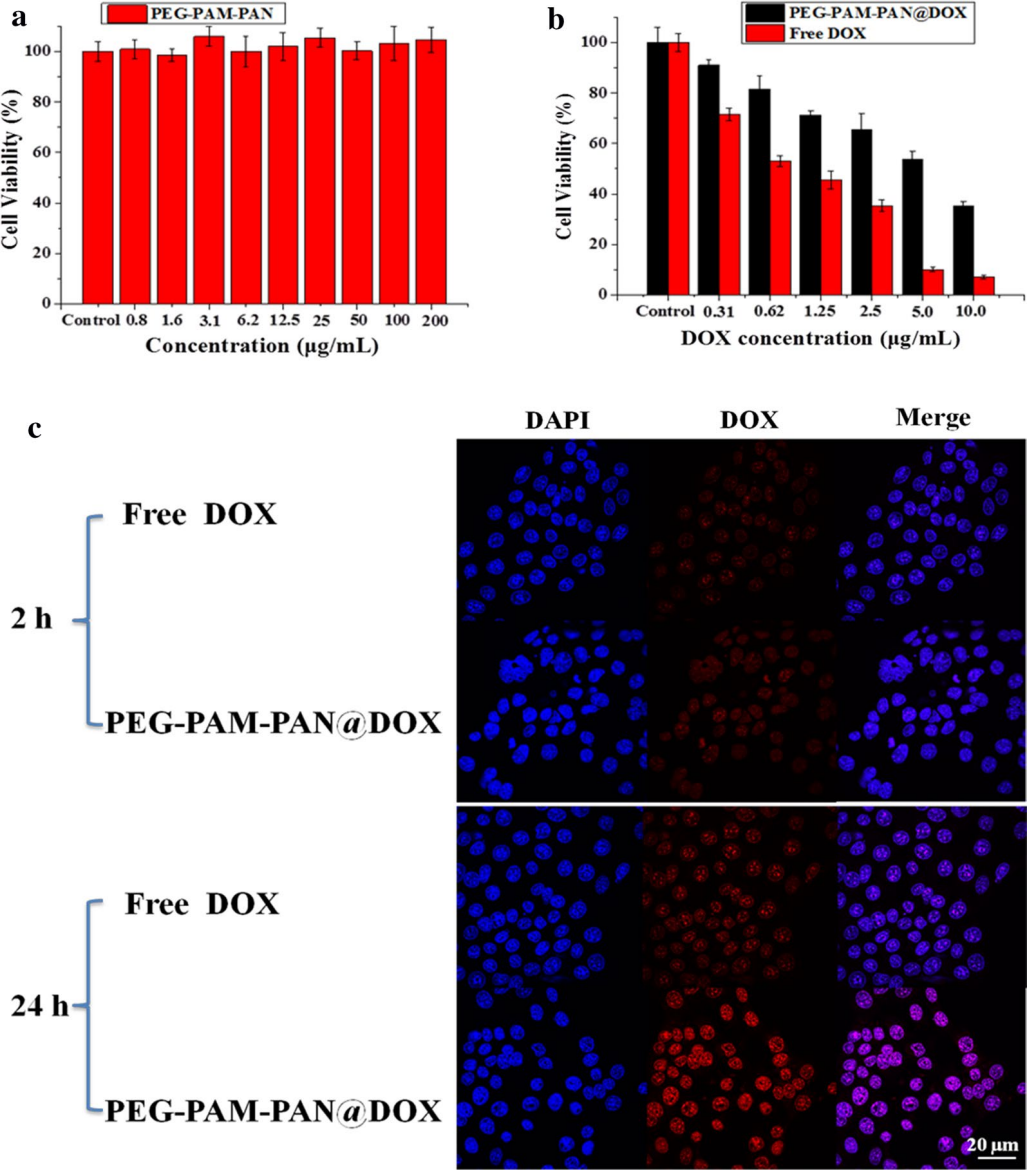
Toxicity and uptaken studies of nanomedicine. **a**, **b** Typical MTT method was used to evaluate the cytotoxicity of PEG-PAM-PAN, free DOX, and PEG-PAM-PAN@DOX. **c** Cellular uptake of free Dox and NPs using confocal microscope at 2 h and 24 h of treatment

The cytotoxicity of PEG-PAM-PAN, free DOX, and PEG-PAM-PAN@DOX was evaluated by the typical MTT assay and determination of the half-maximal inhibitory concentration (IC50) of chemotherapeutic drugs [49]. As shown in Fig. 4a, PEG-PAM-PAN did not show toxicity to the cells even at an extremely high concentration (200 μg/mL), demonstrating the good biocompatibility of these NPs for delivery applications. The viability of MDA-MB-231 breast cells decreased with increasing concentration of the NPs, indicating a dose-dependent effect (Fig. 4b). The IC50 value of PEG-PAM-PAN@DOX was much lower than that of free DOX (mean ~ 0.62 μg/mL vs. ~ 5 μg/mL), indicating that encapsulation of DOX in NPs improved the cytotoxic action of the drug. Cellular uptake of the NPs was evaluated to understand this mechanism based on confocal microscopy observations (Fig. 4c). In the free DOX group, purple fluorescence (overlap of DAPI and DOX) was observed in the MDA-MB-231 nuclei 2 h after treatment, and nuclei with stronger purple fluorescence were observed after 24 h of treatment. For the PEG-PAM-PAN@DOX group, NPs were more efficiently uptaken by the cells, which were evident in the cytoplasm and nucleus with stronger purple fluorescence after 24 h than detected in the free DOX-treated group. These results suggest that the encapsulation of DOX into NPs enhances drug delivery to the cells and increases its cytotoxic effect. Thus, an NP formulation could be used to reduce the systemic toxicity of traditional chemotherapy.

**Fig. 5:**
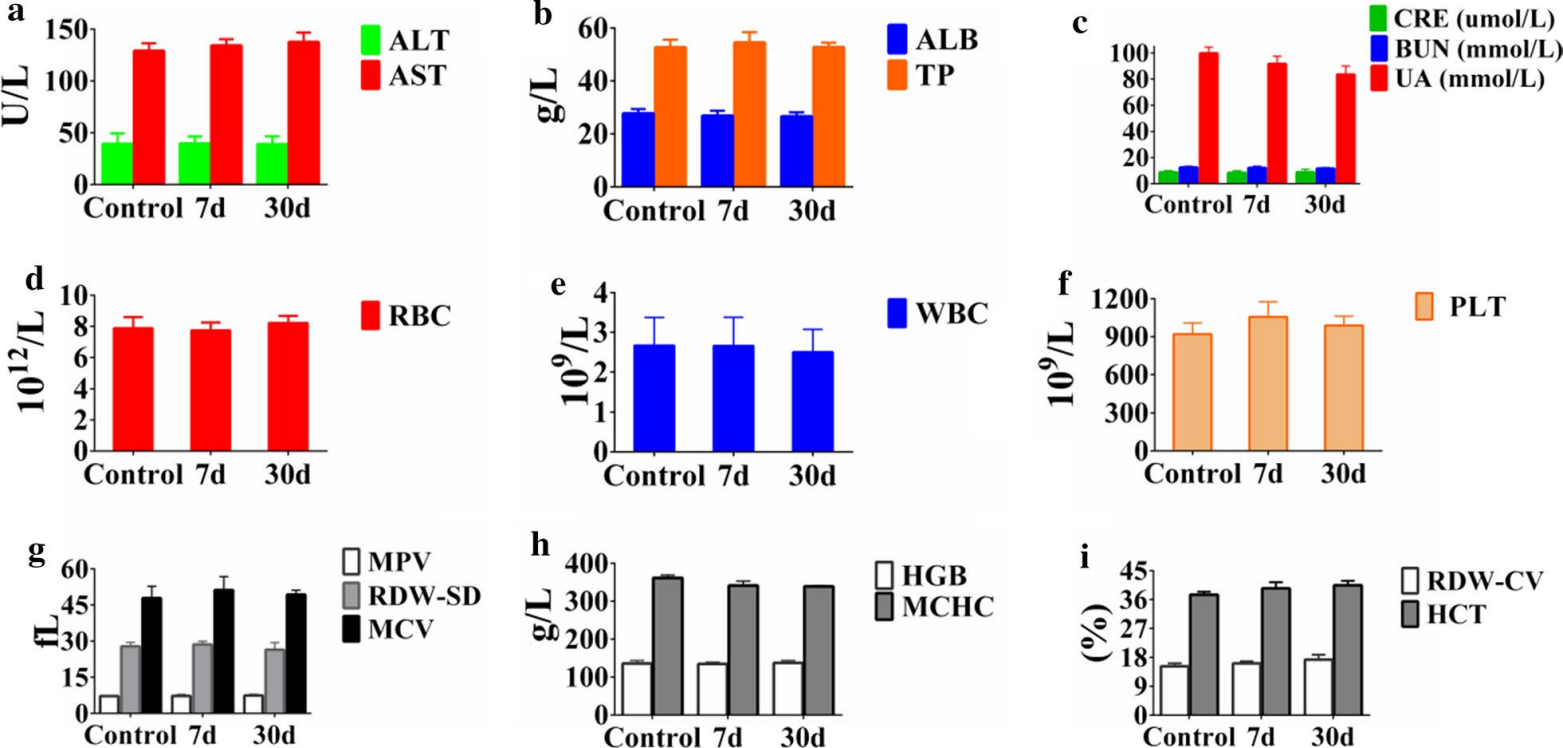
Blood biochemistry data obtained from mice in three groups (control group injected with saline, 7 days and 30 days after administration of PEG-PAM-PAN via tail intravenous injection). **a**, **b** Liver function indicators, **c** kidney function, and **d**–**i** complete blood count

Although the cytotoxicity experiments showed that PEG-PAM-PAN is nontoxic to cells, it was necessary to investigate the potential in vivo toxicity for clinical application. After intravenous injection of PEG-PAM-PAN at 10 mg/kg, no noticeable behavioral abnormality was observed in the mice. There was also no significant difference in the body weight between the mice of group 1 and group 3, indicating that PEG-PAM-PAN was not toxic (Additional file 1: Figure S6). Indicators of liver function (ALT, AST, ALP, and TP) (Fig. 5a, b) and kidney function (CRE, BUN, and UA) (Fig. 5c) were well within the normal ranges for all three groups, which indicated no significant hepatic or kidney dysfunction induced by PEG-PAM-PAN. Similarly, various vital hematology parameters (Fig. 5d–i) including red blood cells, white blood cells, platelets, hemoglobin, mean platelet volume, mean corpuscular volume, mean corpuscular hemoglobin concentration, hematocrit, red blood cell distribution width variation coefficient, and red blood cell distribution width standard deviation, exhibited no significant variation in comparison with those of the control group. Finally, H&E staining of different organs (heart, liver, spleen, lung, and kidney) indicated that PEG-PAM-PAN does not have any appreciable adverse effect on these tissues (Additional file 1: Figure S7). In summary, these in vivo results validated that PEG-PAM-PAN has excellent biocompatibility and can serve as a promising drug nanocarrier.

### In vivo CEST imaging of PEG-PAM-PAN @DOX

**Fig. 6:**
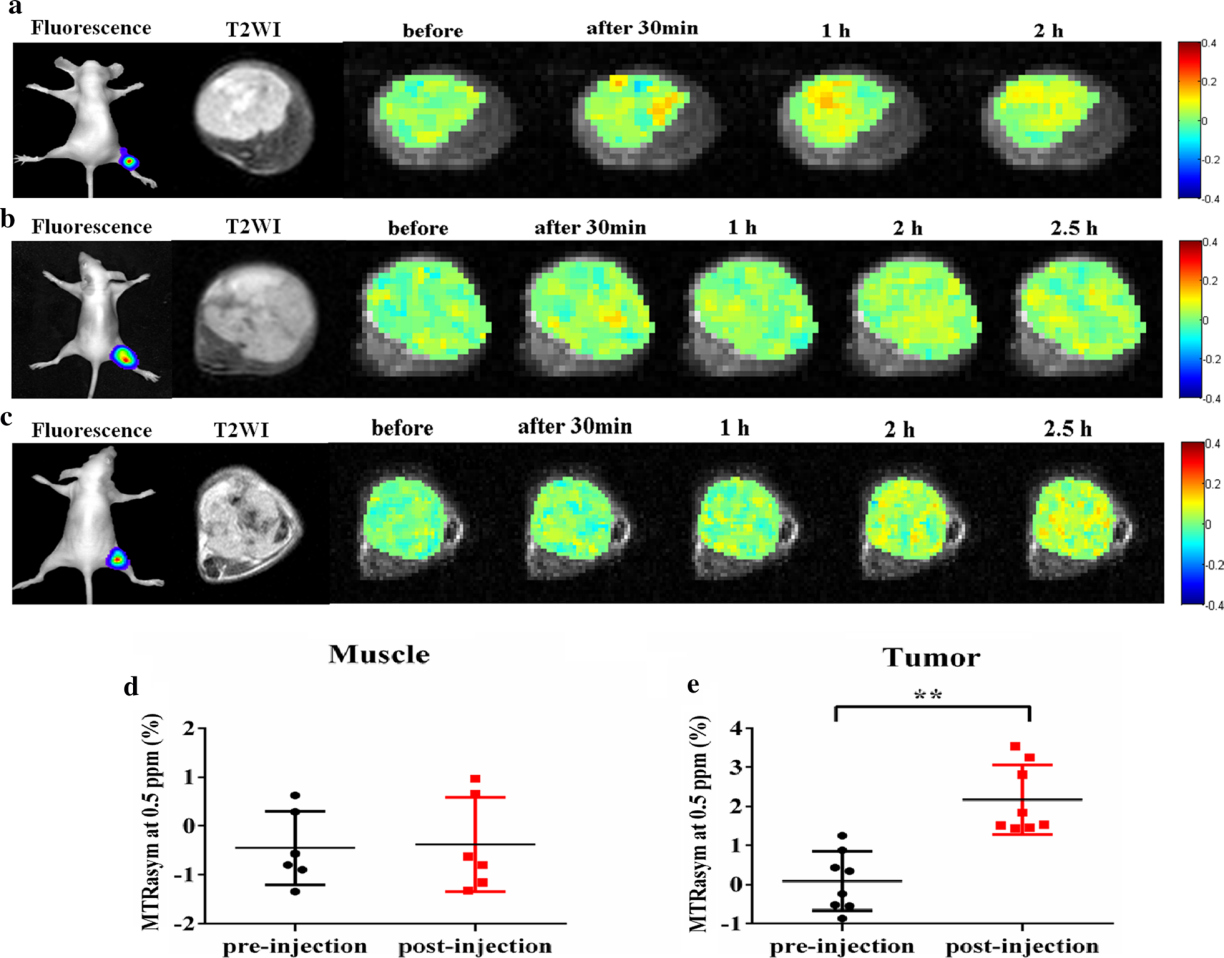
In vivo CEST imaging nanomedicines intravenous injected in mice bearing MDA-MB-231 breast of cancer xenografts. The imaging showed that the nanomedicine was mainly accumulated in tumor areas and peaked at 1 h (**a**, n = 2), 2 h (**b**, n = 4), and 2.5 h (**c**, n = 2) after tail intravenous injection; The relative MTRasym at 0.5 ppm for muscle and entire tumor for the two groups, respectively (**d**, **e**). (**p < 0.01, paired *t* test)

In recent years, significant progress has been made in the design of novel molecular MRI probes, although very few of them have been applied in vivo to date [28]. Moreover, research on imaging approaches that can both noninvasively monitor the drug distribution and evaluate therapeutic features in vivo is limited. Owing to their small size and large number of exchangeable protons, NPs can enter the extracellular space of a tumor via the well-known EPR effect, thereby rendering a higher signal-to-noise ratio (SNR) in tumors [50, 51]. These properties can therefore be exploited for CEST imaging to monitor the accumulation of a nanomedicine at predetermined time intervals in vivo. Pre-injection CEST images were acquired as background for baseline referencing. Compared to the pre-injection baseline images, the CEST signal of PEG-PAM-PAN@DOX contrast (at 0.5 ppm) slightly increased and accumulated in the tumors. The average relative MTRasym determined post-injection 2 h was significantly higher than that measured pre-injection (n = 8, 2.17 ± 0.88% vs. 0. 09 ± 0.75%, *p* < 0.01) (Fig. 6e). No significant difference was found in the relative MTRasym at 0.5 ppm in the muscle, indicating that the increase of CEST contrast is specific to the enhanced accumulation of PEG-PAM-PAN@DOX in tumors (Fig. 6d).

Previous studies [48, 52] have shown that small-molecule agents often have rapid uptake and clearance in both pathologic and normal tissues. Thus, the typical time window for detection is 30 min after administration. In our study, the nanomedicine showed a prolonged detection window of 1 h to 2.5 h after administration (Fig. 6a–c). The plasma half-life of the nanomedicine was most likely prolonged because the hydrophilic ends are not easily recognized by the defense system. In addition, the complexity of the tumor microenvironment (such as the highly heterogeneous vascular anatomy, low extracellular pH, and slow and variable blood flow) might also have contributed to the marked variation in particle delivery [53, 54].

The magnitude of a CEST signal is directly correlated with the saturation power applied during a CEST MRI experiment. In our study, increasing the power produced a larger CEST signal, which facilitated signal detection. Unfortunately, using a higher saturation power not only increases the magnetization transfer (MT) signal dramatically but also increases the likelihood of reaching specific absorption rate (SAR) safety limits [55, 56]. Therefore, a relatively lower saturation power may reduce the effects of MT and is preferred in a clinical setting with regard to safety concerns. The highest MTRasym values were obtained using a relatively low saturation power (1.5 μT), which is a safe level for future in vivo studies. These results are consistent with an earlier optimization report on the CEST quantification technique, which indicated that the selectivity of saturation could be improved using a low saturation power of 0.5–6 μT, and saturation could reach a steady state using a long duration of 1–5 s [47, 48, 57]. In addition, the CEST effect and SNR can be enhanced at higher field strengths [58]. Several studies have shown that there is a fourfold reduction in the variance of the observed CEST or MT effect compared to previous results obtained at 3.0 T [55, 58]. Thus, based on our initial experiences, we performed CEST imaging of the breast tumors in vivo at 7.0 T.

It has been reported that NPs (100–200 nm) allow for achieving 24-fold higher accumulation of therapeutic drugs [59, 60]. Moreover, nanocarriers of an appropriate size (e.g. ~ 50–200 nm) are more likely to accumulate in tumor areas [9]. In our study, the size of the NPs increased from 113.4 to 127.2 nm after embedding DOX, which was still very suitable for drug delivery. The use of an exogenous CEST agent has an advantage of acquiring CEST MRI images both before and after administration of the agent so that the difference between the images can isolate the CEST effect from that of the agent [48]. In addition, NPs can be cleared through biodegradation. Therefore, CEST MRI of the breast holds good promise as a new biomarker to evaluate the effects of PEG-PAM-PAN@DOX treatment owing to its ability of noninvasively detecting changes at the cellular level. This technology may further play a key role in understanding breast tumor progression and response to treatment.

### Chemotherapeutic efficacy of PEG-PAM-PAN @DOX for breast cancer

**Fig. 7:**
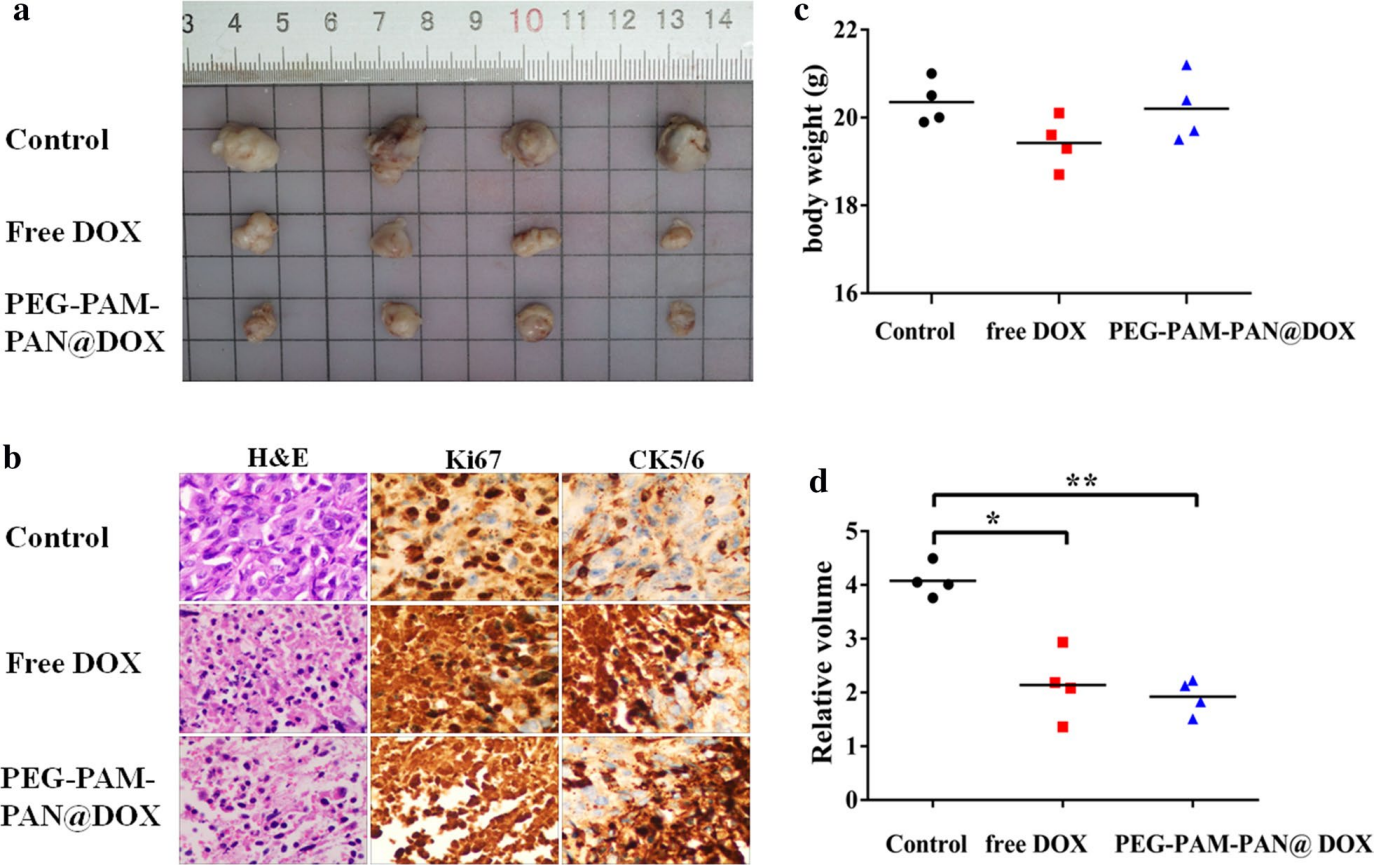
Chemotherapeutic efficacy of different treatments for breast cancer. **a** Comparison of gross morphology for treatment effect assessment; **b** H&E, Ki67, and CK5/6 (40 × 10) staining indicated that there were less proliferative cells but more apoptotic cells in both PEG-PAM-PAN@DOX group and free DOX group; Body weight (**c**) and tumor relative volume (**d**) of mice after different treatments. (*p < 0.05; **p < 0.01; one-way ANOVA)

According to the in vivo CEST imaging results, PEG-PAM-PAN@DOX was speculated to have an antitumor effect in tumor-bearing mice. As shown in Fig. 7a, the gross morphology of the free DOX group and NPs group showed a notable therapeutic effect compared to that of the control group. However, PEG-PAM-PAN@DOX and free DOX appeared to have the same effect of inhibiting the tumor in terms of gross morphology. In theory, PEG-PAM-PAN@DOX could be more effective than free DOX at the same dose. However, upon injection of even the largest doses of PEG-PAM-PAN@DOX in mice, the amount of DOX could not reach the same level as obtained with free DOX. This may be a technical limitation of our experiment. Indeed, the need to improve the drug-loading capacity is a widespread challenge of nanomedicine at present. Thus, further study (such as the use of alternative loading approaches or other small organic agents) for increasing the drug-loading capacity is needed [61, 62]. H&E staining further demonstrated cell necrosis and apoptosis in the tumor tissue after treatment, indicating the effective tumor-suppressing capacity of PEG-PAM-PAN@DOX. Furthermore, immunostaining for Ki67 and CK5/6, as common staining methods for clinical pathological analysis, indicated less proliferative cells but more apoptotic cells in both the PEG-PAM-PAN@DOX group and the free DOX group compared with those of the control group (Fig. 7b). In addition, there was no obvious loss of body weight of the mice in the control group and PEG-PAM-PAN@DOX group, whereas notable body weight reduction was observed in the free DOX group (Fig. 7c). This decrease in body weight was attributed to the known toxicity of DOX in mice. However, this toxicity was reduced with administration of PEG-PAM-PAN@DOX NPs. Thus, PEG-PAM-PAN@DOX NPs may be potentially superior nanocarriers for cancer therapy. The relative tumor volumes of the free DOX group and PEG-PAM-PAN@DOX group were lower than those of the control group, demonstrating a notable therapeutic effect (n = 4; *p* *<* 0.05), and there was no significant difference in the relative tumor volume between the experimental groups (n = 4; *p* > 0.05) (Fig. 7d). Collectively, these results indirectly demonstrate that the nanomedicine could improve the therapeutic effect at the same dose applied in free form with reduced side effects.

## Conclusions

It is feasible to synthesize the novel nanomedicine PEG-PAM-PAN@DOX with CEST effects owing to its self-assembling nature. The synthesis of this biodegradable nanomedicine was effective and straightforward. The cytotoxicity and in vivo toxicity assessment results validated that PEG-PAM-PAN has excellent biocompatibility and can serve as a promising broad-spectrum drug nanocarrier to load a variety of hydrophobic small-molecule drugs on its core for tumor chemotherapy. The CEST MRI results showed that, compared to traditional drug detection in windows (30 min), PEG-PAM-PAN@DOX NPs could prolong the drug exposure time to enhance chemotherapeutic efficacy. Moreover, both in vitro and in vivo experiments proved that PEG-PAM-PAN@DOX can be used not only in CEST imaging at 7.0 T to reflect the pH and to monitor drug accumulation in tumors, but also in cancer therapy. It is particularly relevant for the early evaluation of efficacy and subsequently establishing tailored individualized treatments. Therefore, nanomedicine with CEST imaging to reflect the characterization of tumors and therapeutic functions has great potential medical application.

## Supplementary information

**Additional file 1** CEST imaging of AM at different saturation power and time; structure of PEG-PAM-PAN@DOX; TEM imaging; linear regression equation was calculated from the absorptance of different DOX concentration; the relationship between different saturation power/time and CEST ratio% of PEG-PAM-PAN@DOX; the body weight of mice; H&E staining (**Figures S1**–**S6**).

## Data Availability

All datasets generated for this study are included in the manuscript and its additional files.

## Abbreviations

MTT: methyl thiazolyl tetrazolium
AM: acrylamide
MTRasym: asymmetry in the magnetization transfer ratio
CEST: chemical exchange saturation transfer
CBC: complete blood count
DOX: doxorubicin
DMEM: Dulbecco’s modified Eagle’s medium
DLS: dynamic light scattering
PBS: phosphate-buffered saline
DAPI: 4-6-diamidino-2-phenylindole
IC50: half-maximal inhibitory concentration
H&E: hematoxylin and eosin
MRI: magnetic resonance imaging
PEG: polyethylene glycol
RF: radiofrequency
ST%: saturation transfer efficiency
