# Supplementary material for: Novel nanomedicine with a chemical-exchange saturation transfer effect for breast cancer treatment in vivo
Source: J Nanobiotechnology. 2019 Dec 17;17:123. doi: 10.1186/s12951-019-0557-0 (PMC6918642; doi:10.1186/s12951-019-0557-0)
Supplement: Supplementary file 1 — Additional file 1 CEST imaging of AM at different saturation power and time; structure of PEG-PAM-PAN@DOX; TEM imaging; linear regression equation was calculated from the absorptance of different DOX concentration; the relationship between different saturation power/time and CEST ratio% of PEG-PAM-PAN@DOX; the body weight of mice; H&E staining (Figures S1–S6). [file 12951_2019_557_MOESM1_ESM.doc]

# Additional file

**Novel nanomedicine with** **a chemical-exchange saturation transfer effect for breast cancer treatment *in vivo***

Yanlong Jia^1†^, Chaochao Wang^2†^, Jiehua Zheng^3^, Guisen Lin^1^, Dalong Ni^4^, Zhiwei Shen^1^, Baoxuan Huang^2^, Yan Li^1^, Jitian Guan^1^, Weida Hong^3^, Yuanfeng Chen^1^, and Renhua Wu^1^*****

^1^Department of Radiology, Second Affiliated Hospital, Shantou University Medical College, Shantou 515041, P.R. China

^2^Shanghai Key Laboratory of Advanced Polymeric Materials, School of Chemistry and Molecular Engineering, East China University of Science and Technology, 130 Meilong Road, Shanghai 200237, P.R. China

^3^Department of General Surgery, Second Affiliated Hospital, Shantou University Medical College, Shantou 515041, P.R. China

^4^Departments of Radiology and Medical Physics, University of Wisconsin-Madison, Madison, WI, 53705, USA.

***Correspondence Authors:** [rhwu@stu.edu.cn](mailto:rhwu@stu.edu.cn)

**^†^** Yanlong Jia and Chaochao Wang contributed equally to this work.

.


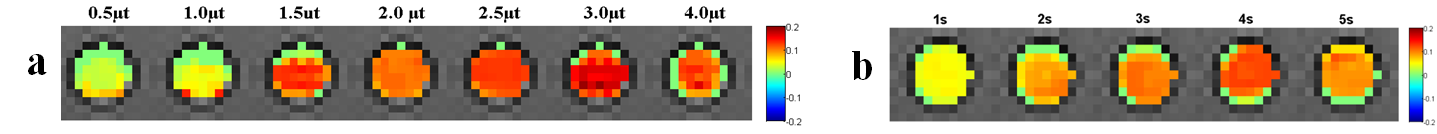


**Figure S1** (**a**) CEST imaging of AM at different power; (**b**) CEST imaging of AM at different saturation time.


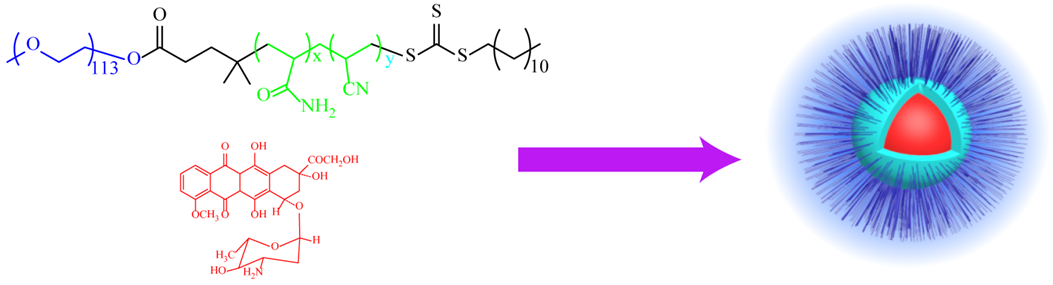


**Figure S2** Structure of PEG-PAM-PAN@DOX and contains many amide groups.


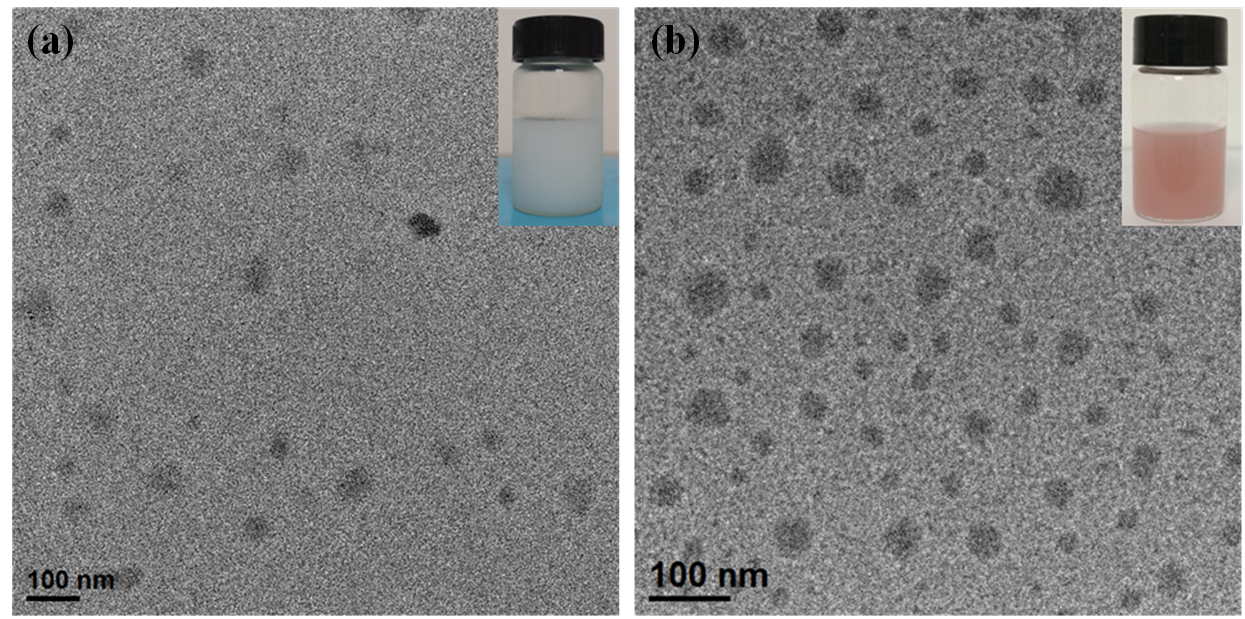


**Figure S****3** TEM image showed that PEG-PAM-PAN (**a**), PEG-PAM-PAN@DOX (**b**) NPs were well-dispersed, spherically shaped particles, which were stable in water. Inset: Photo of as-made PEG-PAM-PAN and PEG-PAM-PAN@DOX in water.


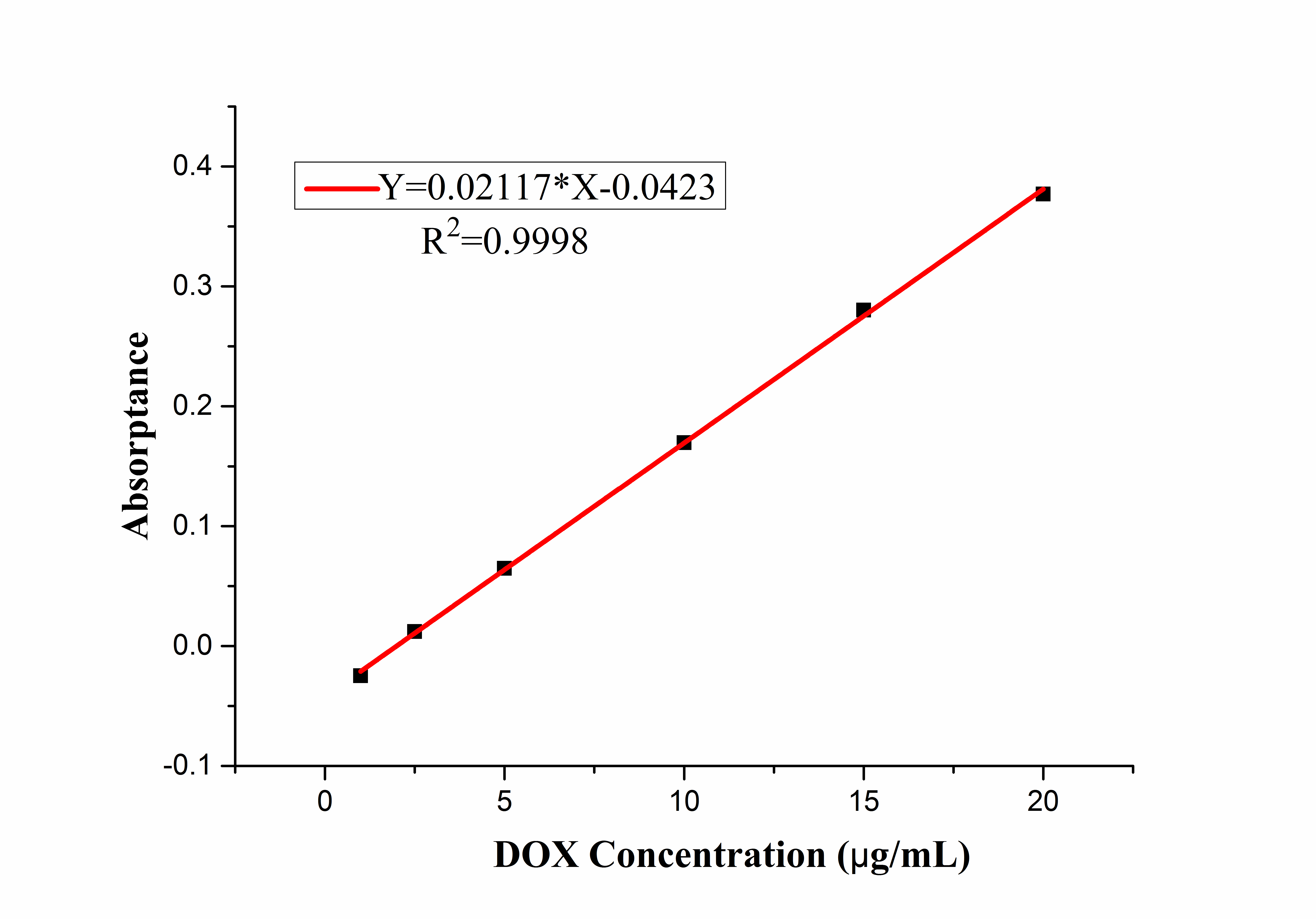


**Figure S4** The linear regression equation was used to calculate the amount of DOX loaded in nanoparticles.


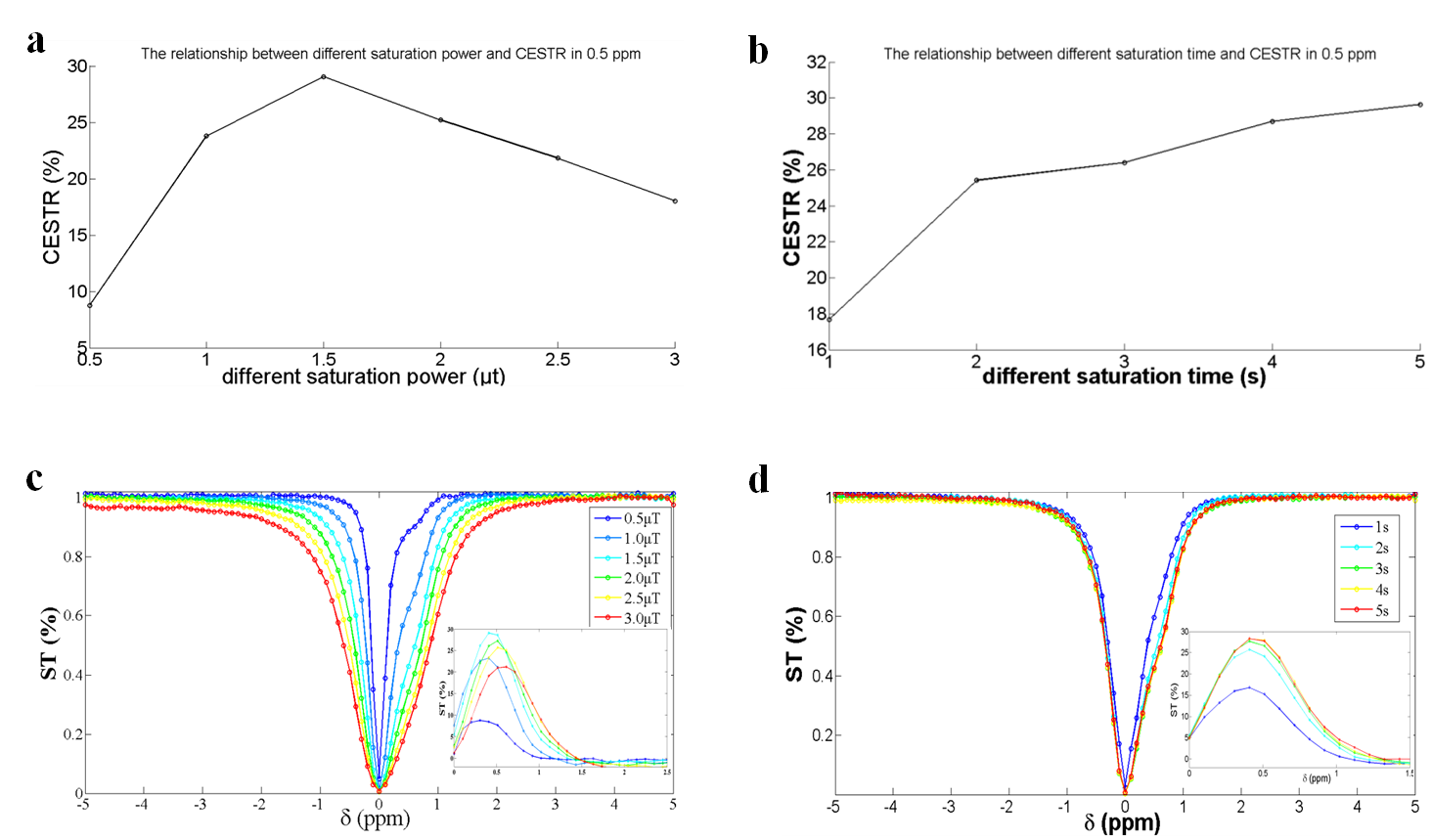


**Figure S5** (**a**) The relationship between different saturation power and CEST ratio% of PEG-PAM-PAN@DOX; (**b**) The relationship between different saturation time and CEST ratio% of PEG-PAM-PAN@DOX; (**c**) Z-spectra of PEG-PAM-PAN@DOX at different saturation power; (**d**) Z-spectra of PEG-PAM-PAN@DOX at different saturation time.


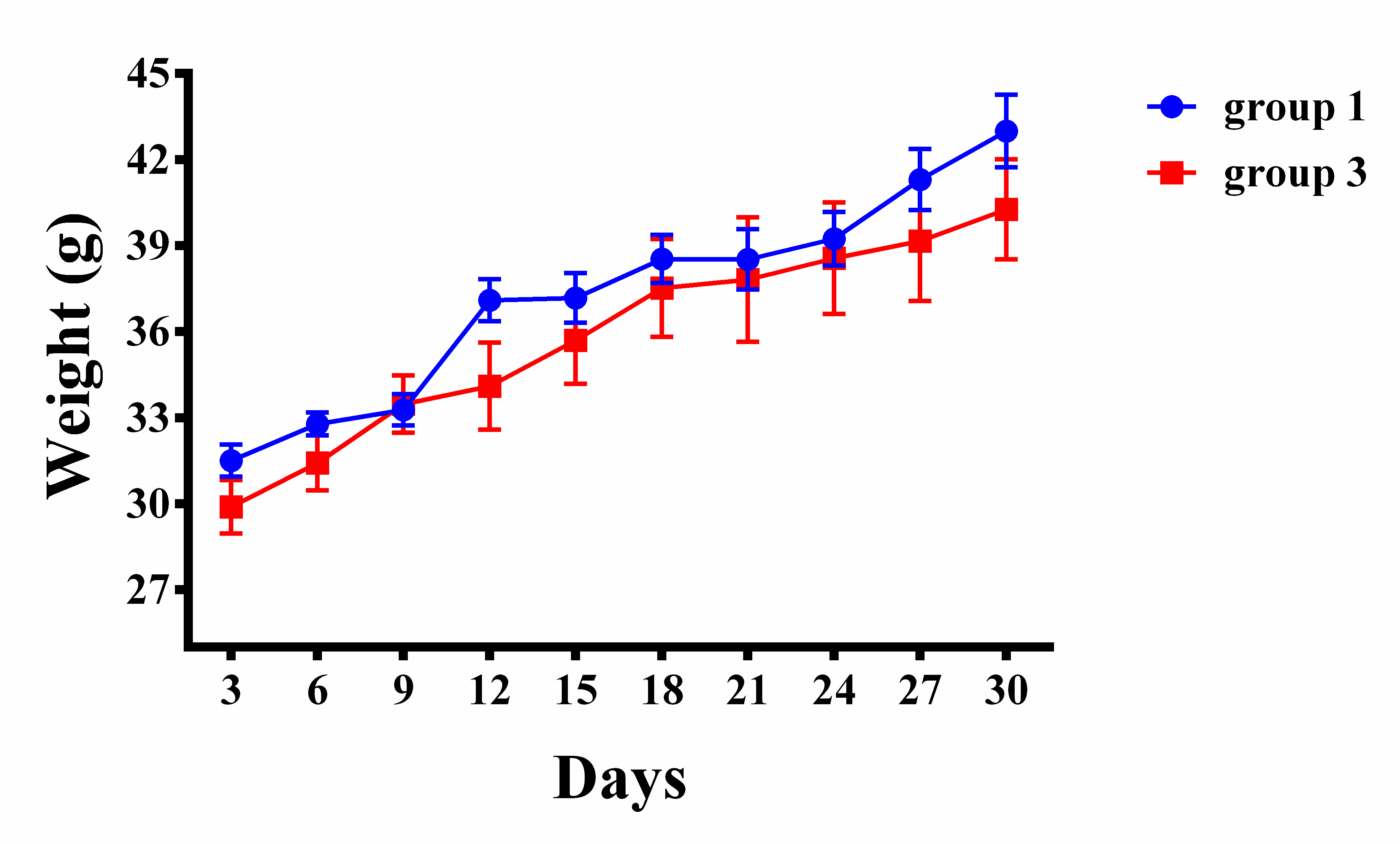


**Figure S6**  There was no significant difference of the body weight between group 1 and group 3, indicating that the PEG-PAM-PAN was no toxic.


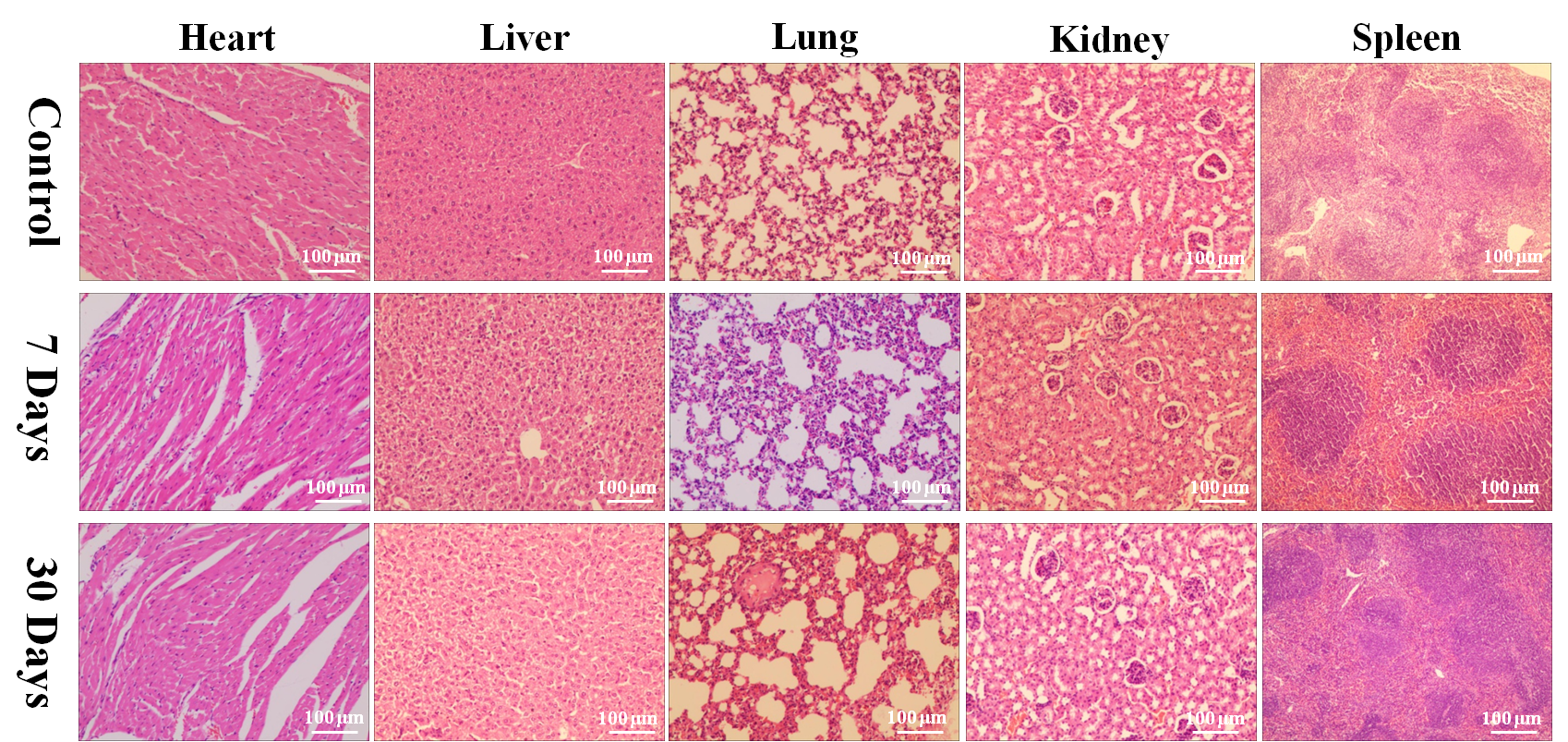


**Figure S7** H&E stain (10×10) of tissues from mice in three groups (control group injected with saline, 7 days and 30 days after administration of PEG-PAM-PAN via tail intravenous injection) to monitor the histological changes in heart, liver, lung, kidney, and spleen.
